# Predictive value of *in vitro* assays depends on the mechanism of toxicity of metal oxide nanoparticles

**DOI:** 10.1186/1743-8977-10-55

**Published:** 2013-10-25

**Authors:** Wan-Seob Cho, Rodger Duffin, Mark Bradley, Ian L Megson, William MacNee, Jong Kwon Lee, Jayoung Jeong, Ken Donaldson

**Affiliations:** 1 ELEGI/Colt Laboratory, Centre for Inflammation Research, University of Edinburgh, 47 Little France Crescent, Edinburgh EH16 4TJ, UK; 2 Department of Medicinal Biotechnology, College of Natural Resources and Life Science, Dong-A University, Busan 604-714, Republic of Korea; 3 School of Chemistry, West Mains Road, University of Edinburgh, Edinburgh, UK; 4 Free Radical Research Facility, Department of Diabetes & Cardiovascular Science, University of the Highlands & Islands, Centre for Health Science, Inverness, UK; 5 Department of Toxicological Research, National Institute of Food and Drug Safety Evaluation, Ministry of Food and Drug Safety, Osong 363-700, Republic of Korea

**Keywords:** *In vitro*, *In vivo*, Inflammation, Mechanism, Nanoparticles, Prediction, Toxicity

## Abstract

**Background:**

Hazard identification for risk assessment of nanoparticles (NPs) is mainly composed of *in vitro* cell-based assays and *in vivo* animal experimentation. The rapidly increasing number and functionalizations of NPs makes *in vivo* toxicity tests undesirable on both ethical and financial grounds, creating an urgent need for development of *in vitro* cell-based assays that accurately predict *in vivo* toxicity and facilitate safe nanotechnology.

**Methods:**

In this study, we used 9 different NPs (CeO_2_, TiO_2_, carbon black, SiO_2_, NiO, Co_3_O_4_, Cr_2_O_3_, CuO, and ZnO). As an *in vivo* toxicity endpoint, the acute lung inflammogenicity in a rat instillation model was compared with the *in vitro* toxicity endpoints comprising cytotoxicity, pro-inflammatory cytokine expression, or haemolytic potential. For *in vitro* assays, 8 different cell-based assays were used including epithelial cells, monocytic/macrophage cells, human erythrocytes, and combined culture.

**Results:**

ZnO and CuO NPs acting via soluble toxic ions showed positive results in most of assays and were consistent with the lung inflammation data. When compared in *in vitro* assays at the same surface area dose (30 cm^2^/mL), NPs that were low solubility and therefore acting via surface reactivity had no convincing activity, except for CeO_2_ NP. Cytotoxicity in differentiated peripheral blood mononuclear cells was the most accurate showing 89% accuracy and 11% false negativity in predicting acute lung inflammogenicity. However, the haemolysis assay showed 100% consistency with the lung inflammation if any dose, having statistical significance was considered positivity. Other cell-based *in vitro* assays showed a poorer correlation with *in vivo* inflammogenicity.

**Conclusions:**

Based on the toxicity mechanisms of NPs, two different approaches can be applied for prediction of *in vivo* lung inflammogenicity. Most *in vitro* assays were good at detecting NPs that act via soluble ions (i.e., ZnO and CuO NP). However, *in vitro* assays were limited in detecting NPs acting via surface reactivity as their mechanism of toxicity, except for the haemolysis assay.

## Background

Metal oxide nanoparticles (NPs) have been used in various applications including industrial, electrical, pharmaceutical, and biomedical fields because of their unique physicochemical properties compared to bulk chemicals [1]. The high production volume of NPs and increasing numbers of functionalized versions constitutes a burden for toxicity testing and risk assessment for NP exposures [2]. In response to increasing concerns about the safety of manufactured nanomaterials, OECD (Organisation for Economic Co-operation and Development) launched an internationally harmonised programme about hazard, exposure, and risk assessment of nanomaterials in 2006 [3]. After six years of work, the OECD has come to conclusion that the current testing approaches are generally acceptable for nanomaterials although some adoptions may be necessary for the certain Test Guidelines [4].

Current hazard identification for risk assessment of NPs is mainly conducted with the aid of both *in vivo* and *in vitro* toxicity approaches. *In vivo* animal experimentation is more informative than *in vitro* experimentation, but there are major ethical and financial limitations to the *in vivo* approach [5]. Therefore, *in vitro* assay have been suggested as an alternative method to *in vivo* testing; indeed *in vitro* experiments are often used as an initial screen for the toxicity of substances and evaluation of their toxic mechanisms. There are several *in vitro* cell-based testing methods that are frequently used in this setting, including some assays which combine several cell types [6], and others that use differentiated macrophages from monocytic cells, which are more sensitive to weak stimuli [7]. However, in recent studies comparing several cell lines, cells were found to respond differently to NPs depending on the physiological functions and activities of cells [8,9] and failed to predict the *in vivo* lung inflammogenicity [10]. However, those studies were performed with a limited number of particles and cell assays; more profound studies are essential to correlate *in vitro* and *in vivo* toxicity assays.

There are several critical factors that can produce discrepancies between studies. Dose is one of the most important factors when comparing several *in vitro* assays with *in vivo* assays, because even NPs with low toxicity can be toxic at high doses [11]. Surface area has been suggested as an appropriate dose metric in nanotoxicology rather than mass [12]. In addition to surface area metric, the dispersion of NPs is very important because highly agglomerated NPs showed less toxicity or inflammogenicity compared to well-dispersed NPs [13]. Determining the target organ for NP exposure is also important for selection of appropriate cell types for *in vitro* assays.

In this study, we were concerned with inhalation exposure of metal oxide NPs in occupational or consumer settings and we therefore used an acute lung inflammation model by intratracheal instillation into the lungs of rats. By comparison, we chose to investigate the nanotoxicity of the same panel of NPs in lung alveolar and bronchial epithelial cells and monocytic or macrophage cells to mimic the *in vivo* lung environment, using a range of *in vitro* toxicity tests.

## Results

### Characterization of NPs

Table 1 summarize the characterization of NPs including primary size, hydrodynamic size, and surface area. All NPs in PBS formed agglomerates, which required sonication to be dispersed. However, inclusion of 5% fetal bovine serum in the PBS, particles formed smaller agglomerates that were readily dispersed because of the protein corona on the surface of NPs. All NPs and FBS showed endotoxin levels below the detection limit.

**Table 1:** Physicochemical properties of the panel of nanoparticles

**NP**^**a**^	**Primary size (nm)**^**b**^	**Surface area (m**^**2**^**/g)**^**c**^	**Hydrodynamic size (nm) in 5% FBS**	**Mass (μg) for 30 cm**^**2**^
CeO_2_	9.7 ± 0.4	24.1	69.3 ± 8.0	125
TiO_2_-rutile	30.5 ± 1.8	27.5	89.9 ± 13.1	109
CB	15.3 ± 6.0	254	45.1 ± 9.8	11.8
SiO_2_	6.2 ± 0.4	523.4	398.1 ± 24.8	5.0
NiO	5.3 ± 0.4	91.8	100.8 ± 38.5	32.7
Co_3_O_4_	18.4 ± 0.8	35.8	148.2 ± 95.5	83.8
Cr_2_O_3_	205 ± 20.7	74.42	195.3 ± 101.4	40.3
ZnO	10.7 ± 0.7	48.2	492.8 ± 25.2	62.0
CuO	23.1 ± 1.0	29	95.6 ± 1.6	103

^a^NPs were provided by Nanostructural and Amorphous Materials Inc. (Houston, TX, USA) except for CB (Evonik Degussa GmbH, Frankfurt, Germany), ZnO (NanoScale Corporation, Manhattan, KS, USA), and CuO (Sigma-Aldrich).

^b^Primary particle sizes were measured by a transmission electron microscopy.

^c^Surface area was measured by BET (Brunauer-Emmett-Teller) method.

### A549 cells

**Figure 1:**
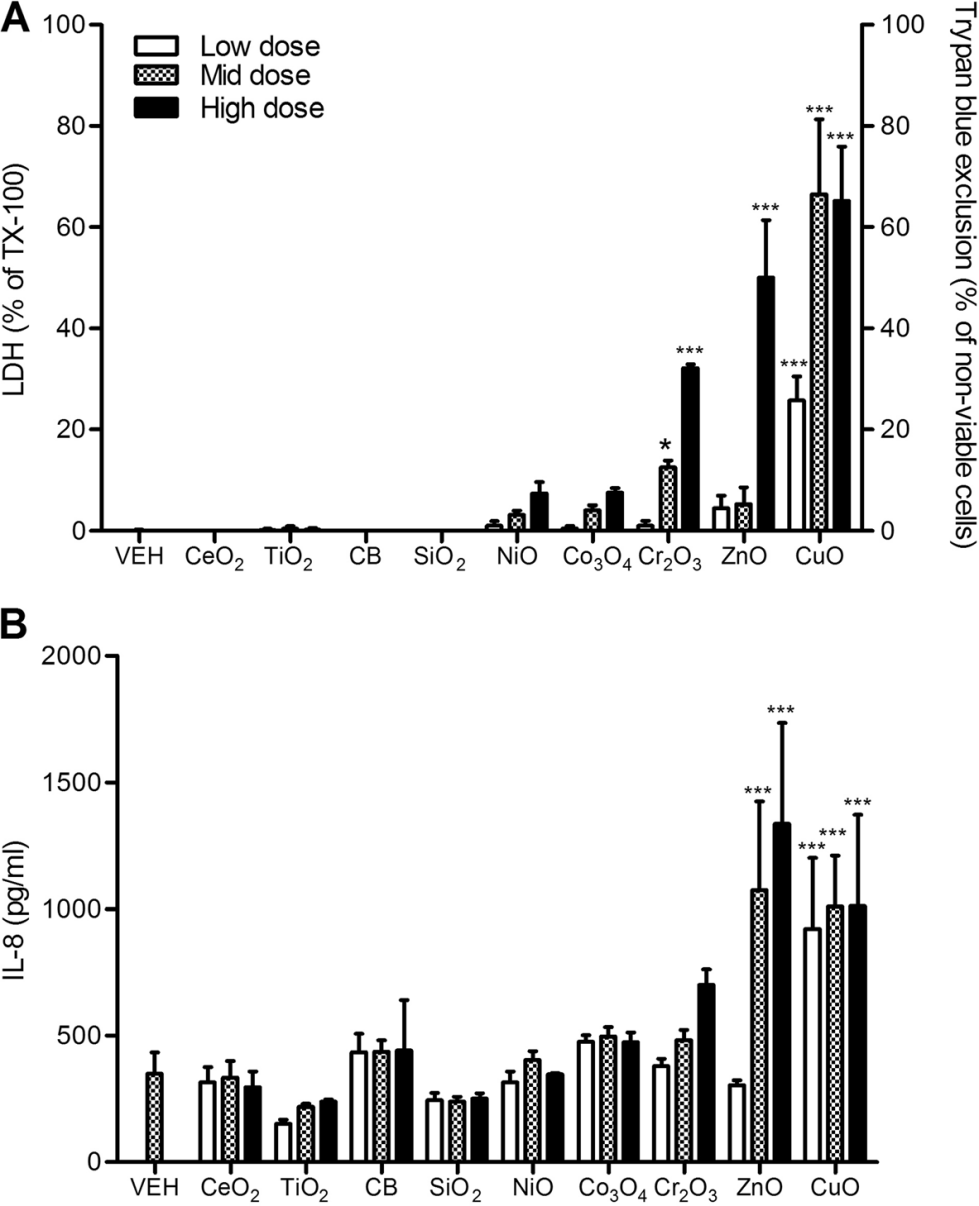
**Cytotoxicity and IL-8 expression of A549 cells after exposure to NPs for 24 h. (A)** Cytotoxicity was measured by trypan blue exclusion for ZnO and CuO NP whilst others were measured by LDH. **(B)** Levels of IL-8 of A549 cells at 24 h following treatment. Note that the surface area doses were 30, 100, and 300 cm^2^/mL except for ZnO and CuO NP which were 3, 10, and 30 cm^2^/mL. Values are mean ± SD from minimum four independent experiments. Significance versus vehicle control (VEH): ^*^p < 0.05, ^***^p < 0.001.

Cr_2_O_3_, ZnO, and CuO NPs showed significant cytotoxicity (cell death measured by LDH levels or trypan blue staining) compared with vehicle control whilst other NPs had little or no toxic effect in this assay at the doses tested (30–300 cm^2^/mL for most NPs, 3–30 cm^2^/mL for ZnO and CuO; Figure 1A). The levels of IL-8 proteins in the supernatant were significantly increased by both ZnO and CuO NP (Figure 1B). Note that ZnO and CuO NP were the most cytotoxic and stimulatory for IL-8 although they were used at doses 10-fold less than the other NPs.

### 16-HBE cells

**Figure 2:**
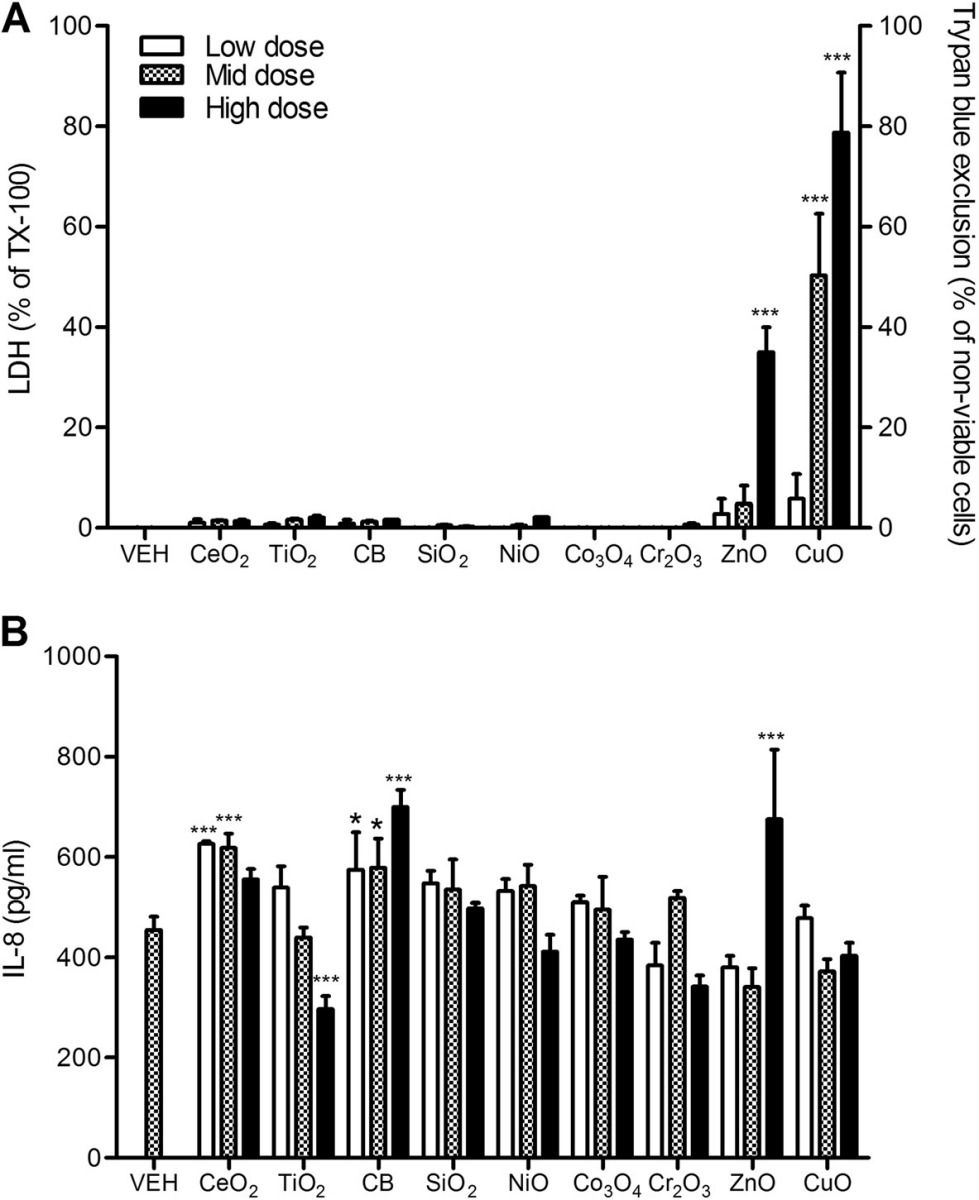
**Cytotoxicity and IL-8 expression of 16-HBE cells after exposure to NPs for 24 h. (A)** Cytotoxicity was measured by trypan blue exclusion for ZnO and CuO NP whilst others were measured by LDH. **(B)** Levels of IL-8 of 16-HBE cells at 24 h following treatment. Note that the surface area doses were 30, 100, and 300 cm^2^/mL except for ZnO and CuO NP which were 3, 10, and 30 cm^2^/mL. Values are mean ± SD from minimum four independent experiments. Significance versus vehicle control (VEH): ^*^p < 0.05, ^***^p < 0.001.

Only ZnO and CuO NP exposure significantly increased cytotoxicity to 16-HBE cells (Figure 2A). The levels of IL-8 proteins were significantly increased by CeO_2_, carbon black (CB), and ZnO NP and significantly decreased by TiO_2_ NP at the high dose (Figure 2B).

### Monocytic THP-1 cells

**Figure 3:**
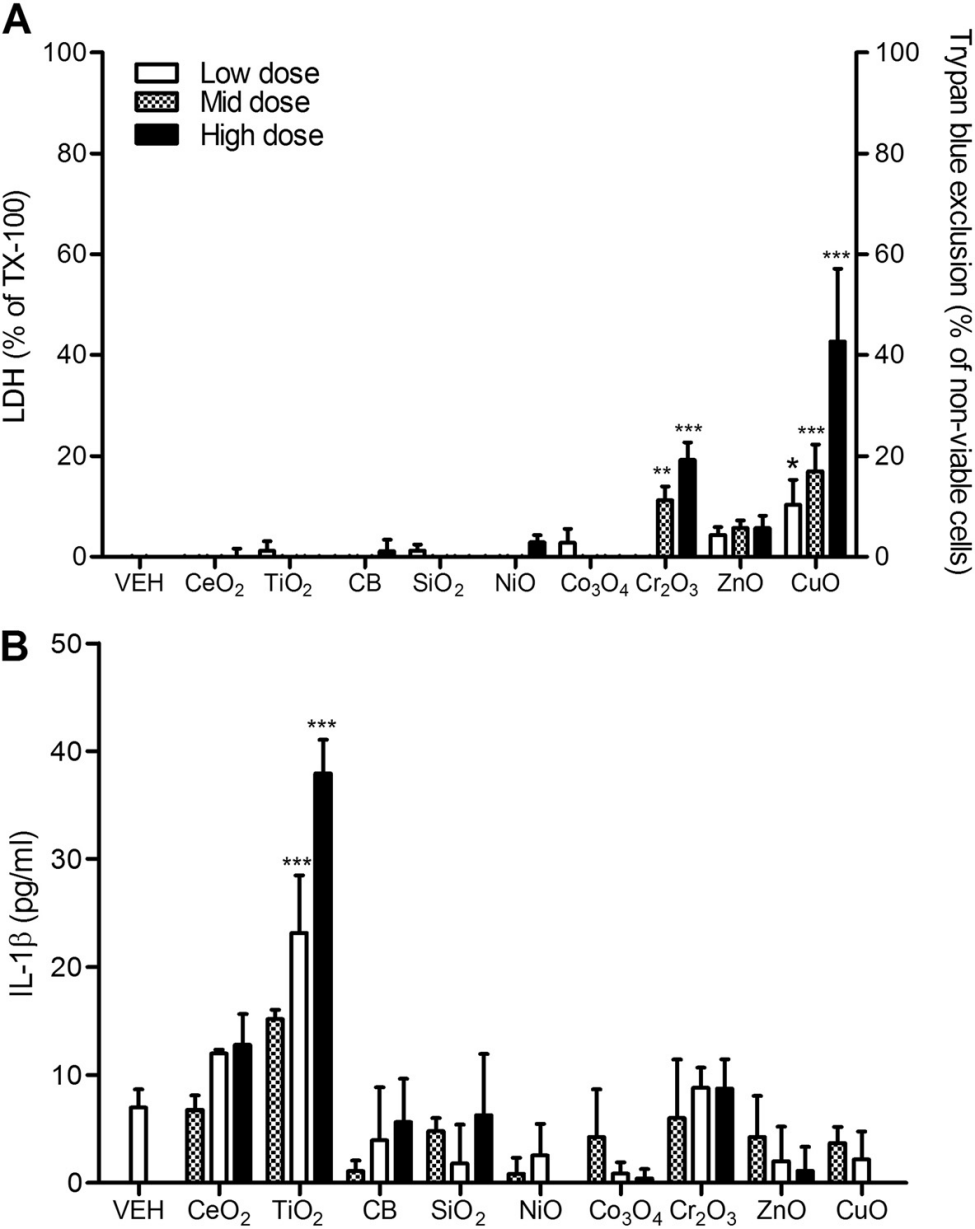
**Cytotoxicity and IL-1β expression of monocytic THP-1 cells after exposure to NPs for 24 h. (A)** Cytotoxicity was measured by trypan blue exclusion for ZnO and CuO NP whilst others were measured by LDH. **(B)** Levels of IL-1β of monocytic THP-1 cells at 24 h following treatment. Note that the surface area doses were 30, 100, and 300 cm^2^/mL except for ZnO and CuO NP which were 3, 10, and 30 cm^2^/mL. Values are mean ± SD from minimum four independent experiments. Significance versus vehicle control (VEH): ^*^p < 0.05, ^**^p < 0.01, ^***^p < 0.001.

Cr_2_O_3_ and CuO NP exposure significantly increased cytotoxicity, whilst other NPs showed no differences compared to vehicle control (Figure 3A). The levels of IL-1β protein significantly increased only with TiO_2_ NP treatment (Figure 3B).

### Alveolar macrophages

**Figure 4:**
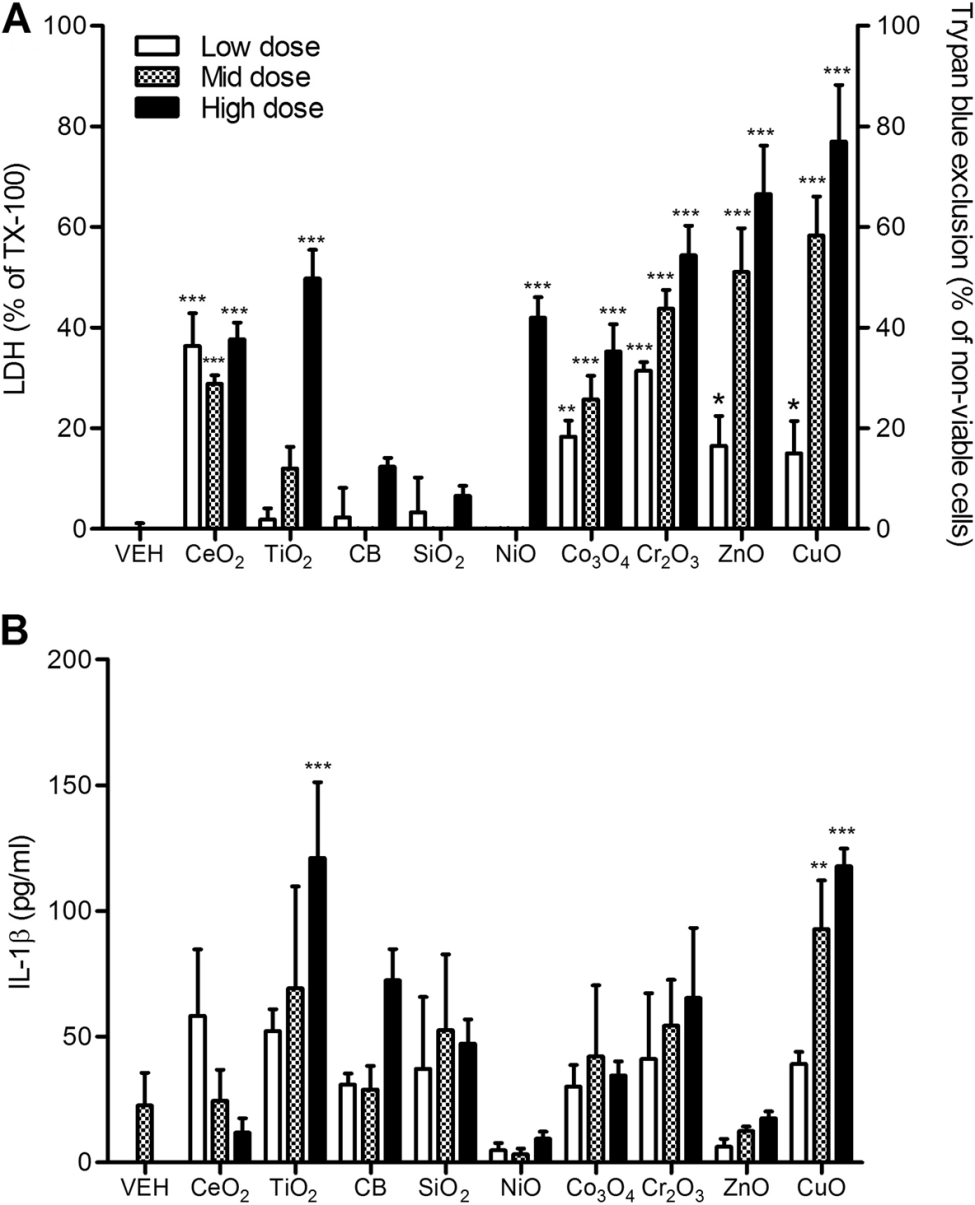
**Cytotoxicity and IL-1β expression of primary cultured alveolar macrophages after exposure to NPs for 24 h. (A)** Cytotoxicity was measured by trypan blue exclusion for ZnO and CuO NP whilst others were measured by LDH. **(B)** Levels of IL-1β of primary cultured alveolar macrophages at 24 h following treatment. Note that the surface area doses were 30, 100, and 300 cm^2^/mL except for ZnO and CuO NP which were 3, 10, and 30 cm^2^/mL. Values are mean ± SD from minimum four independent experiments. Significance versus vehicle control (VEH): ^*^p < 0.05, ^**^p < 0.01, ^***^p < 0.001.

Rat alveolar macrophages showed greater sensitivity to NPs compared to cell lines. All NPs excluding CB and SiO_2_ showed significant cytotoxicity compared to vehicle control (Figure 4A). In comparison with cytotoxicity, the levels of IL-1β showed marginal responses which only TiO_2_ and CuO NP showing significant increases (Figure 4B), although it should be noted that the TiO_2_ NP effect was produced at 300 cm^2^/mL whilst the dose of 10 cm^2^/mL of CuO NP was approximately equipotent for IL-1β.

### Peripheral blood monocyte-derived macrophages (PBMDM)

**Figure 5:**
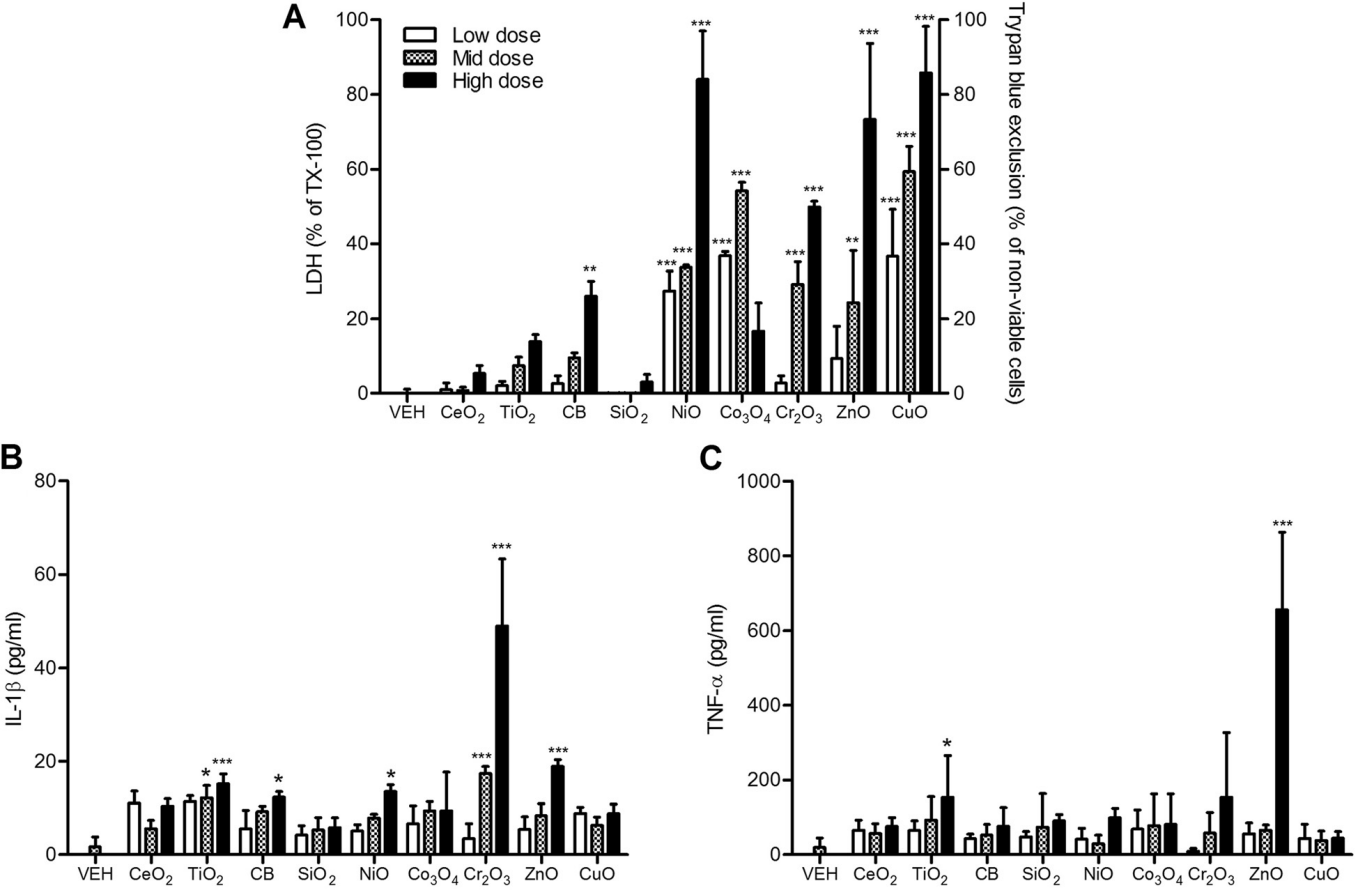
**Cytotoxicity and pro-inflammatory cytokine expression of differentiated PBMC after exposure to NPs for 24 h. (A)** Cytotoxicity was measured by trypan blue exclusion for ZnO and CuO NP whilst others were measured by LDH. **(B)** Levels of IL-1β; **(C)** levels of TNF-α. PBMC were differentiated by 5-day incubation. Note that the surface area doses were 30, 100, and 300 cm^2^/mL except for ZnO and CuO NP which were 3, 10, and 30 cm^2^/mL. Values are mean ± SD from minimum four independent experiments. Significance versus vehicle control (VEH): ^*^p < 0.05, ^**^p < 0.01, ^***^p < 0.001.

PBMDM were differentiated from the peripheral blood mononuclear cells (PBMC) by culturing for 5 days. All NPs excluding CeO_2_, TiO_2_, and SiO_2_ NP showed significant cytotoxicity compared to vehicle control (Figure 5A). The levels of IL-1β were significantly increased by TiO_2_, CB, NiO, Cr_2_O_3_, and ZnO NP (Figure 5B). The levels of TNF-α were significantly increased by TiO_2_ and ZnO NP whilst other NPs were comparable with vehicle control (Figure 5C).

### Differentiated THP-1 cells

**Figure 6:**
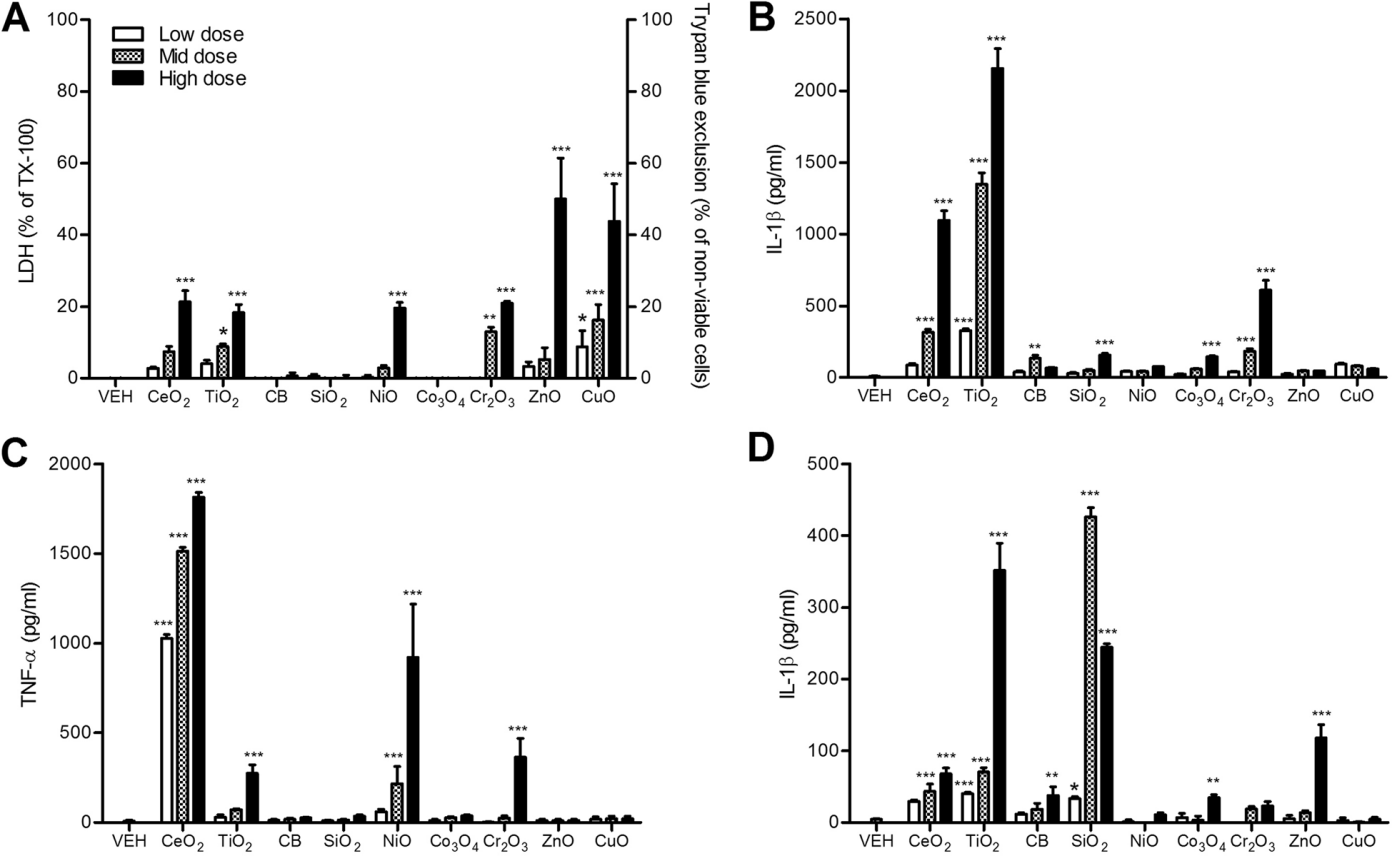
**Cytotoxicity and pro-inflammatory cytokine expression of differentiated THP-1 cells after exposure to NPs for 24 h. (A)** Cytotoxicity was measured by trypan blue exclusion for ZnO and CuO NP whilst others were measured by LDH. **(B)** Levels of IL-1β; **(C)** levels of TNF-α. **(D)** Treatment of NP with cytochalasin D (0.2 μM) showed marked decrease of IL-1β expression compared to NP without cytochalasin D **(B)**. THP-1 cells were differentiated by treatment with PMA (10 ng/mL) and NPs were treated at 30, 100, and 300 cm^2^/mL except for ZnO NP (3, 10, and 30 cm^2^/mL) and CuO NP (0.3, 1, and 3 cm^2^/mL). Values are mean ± SD from minimum four independent experiments. Significance versus vehicle control (VEH): ^*^p < 0.05, ^**^p < 0.01, ^***^p < 0.001.

Differentiated THP-1 cells by treatment with phorbol myristate acetate (PMA) showed much greater sensitivity compared to monocytic THP-1 cells. CeO_2_, TiO_2_, NiO, Cr_2_O_3_, ZnO, and CuO NP showed significantly increased cytotoxicity compared to vehicle control (Figure 6A). The use of differential doses, however, again revealed that ZnO and CuO NP were an order of magnitude or more cytotoxic than the others. Levels of IL-1β were increased in CeO_2_ and TiO_2_ NP-treated cells and showed modest increases with CB, SiO_2_, Co_3_O_4_, and Cr_2_O_3_ NP (Figure 6B). In contrast, the levels of TNF-α showed marked increases on treatment with CeO_2_ and NiO NP and slight increases by TiO_2_ and Cr_2_O_3_ NP (Figure 6C). Treatment of cytochalasin D, a well characterized inhibitor of phagocytosis, dramatically inhibited the expression of IL-1β showing around 1/15 and 1/7 reductions for CeO_2_ and TiO_2_ NP compared to the same treatment without cytochalasin D (Figures 6D).

### Combined culture

**Figure 7:**
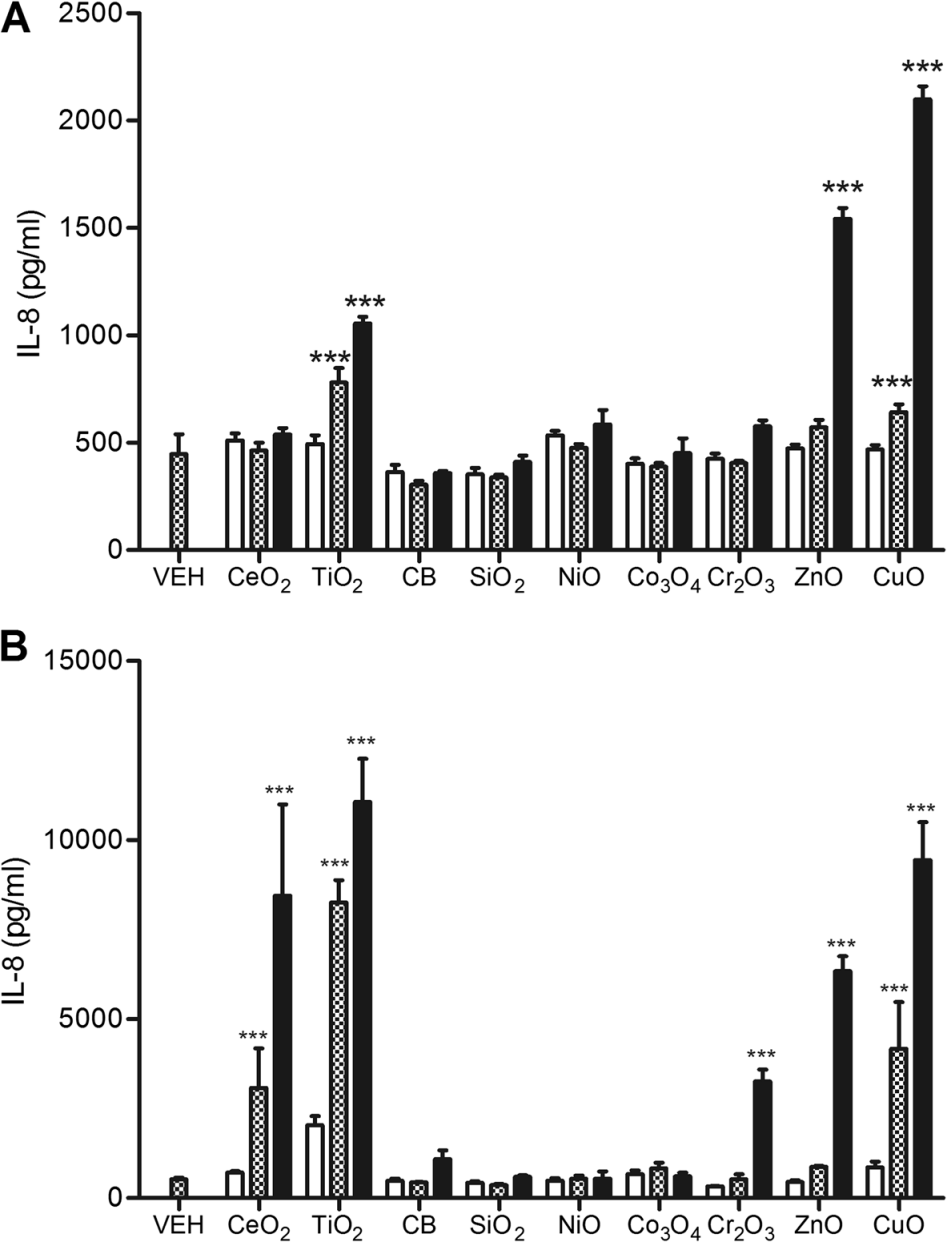
**IL-8 expression by NP-conditioned media.** A549 cells were exposed to conditioned media from NP-exposed differentiated THP-1 cells for 4 h. THP-1 cells were differentiated by treatment for PMA (10 ng/mL) and NPs were treated at surface area doses of 30, 100, and 300 cm^2^/mL except for ZnO NP (3, 10, and 30 cm^2^/mL) and CuO NP (0.3, 1, and 3 cm^2^/mL). **(A)** The levels of IL-8 in the conditioned media from THP-1 cells before addition of A549 cells. **(B)** The levels of IL-8 after addition of NP-free conditioned media to A549 cells. Values are mean ± SD from minimum four independent experiments. Significance versus vehicle control (VEH): ^***^p < 0.001.

The levels of IL-8 in the conditioned media from THP-1 cells before addition of A549 cells were significantly increased by TiO_2_, ZnO, and CuO NP treatment (Figure 7A). Addition of NP-free conditioned media to A549 cells produced a marked increase in IL-8 levels in the CeO_2_, TiO_2_, Cr_2_O_3_, ZnO, and CuO NP treatment groups (Figure 7B). Compared with single treatment to A549 cells presented in Figure 1B, conditioned medium produced more sensitive and higher responses for the IL-8 production.

### Haemolysis assay

**Figure 8:**
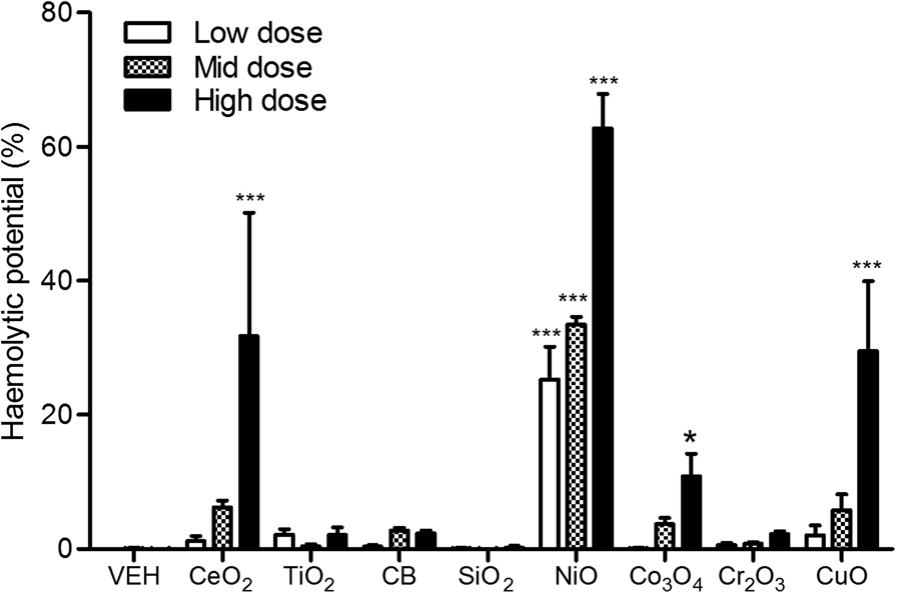
**Haemolysis assay of NP.** NPs were treated at surface area doses of 30, 100, and 300 cm^2^/mL. Values are mean ± SD from minimum four independent experiments. Significance versus vehicle control (VEH): ^*^p < 0.05, ^***^p < 0.001.

CeO_2_, NiO, Co_3_O_4_, and CuO NP showed significant haemolytic potential compared to vehicle control (Figure 8) and all were tested at equal surface area dose. However, it was notable that there was a real difference in potency with NiO NP being at least 2 times more potent than the others in all treatment doses.

### Correlation of *in vitro* assays with *in vivo* acute lung inflammogenicity

Table 2 summarise the number of total granulocytes in the bronchoalveolar lavage fluid in the lungs of rats treated at 150 cm^2^/rat after 24 h of instillation. Table 3 summarise the utility of the various *in vitro* assays for predicting lung inflammogenicity of NP panel. Because the gold standard for comparison between lung inflammation and several *in vitro* assays was dosed at equal surface area dose, we took the 30 cm^2^/mL dose as the measure of a significant effect. Cytotoxicity in differentiated PBMC (PBMDM) showed the best accuracy - 89% (8/9) accuracy, 11% (1/9) false negativity, and 0% (0/9) false positivity to predict acute lung inflammogenicity. The second best assay was cytotoxicity using primary cultured alveolar macrophages which showed 78% (7/9) accuracy, 11% (1/9) false negativity, and 11% (1/9) false positivity. Data from other cell types or pro-inflammatory cytokine assays using PBMDM and alveolar macrophages showed a poor correlation with *in vivo* lung inflammogenicity. In contrast, the *in vitro* assay data at any dose that have statistical significance showed that the haemolysis assay was the most accurate (Table 4). Table 3 can also suggest the mechanisms of action of various metal oxide NPs. Among the NP that caused inflammation, CeO_2_ NP had a range of activities causing IL-8 release from 16-HBE, toxicity to alveolar macrophages, and TNF-α release from THP-1 cells. NiO NP show no persuasive effects whilst Co_3_O_4_ NP seemed to kill macrophages without stimulating them. TiO_2_, CB, SiO_2_, and Cr_2_O_3_ NP showed no convincing effects whilst ZnO and CuO NP caused significant changes in most of assays.

**Table 2:** The number of total granulocytes in the BAL at 24 h after instillation of NPs into rats

**NP**	**Total cells (×10**^**5**^**)**	**Granulocytes (×10**^**5**^**)**
Vehicle control	7.5 ± 3.1	0.2 ± 0.1
CeO_2_	42.3 ± 25.9**	37.1 ± 23.2***
TiO_2_-rutile	7.6 ± 2.6	1.5 ± 1.8
CB	13.1 ± 4.4	8.0 ± 3.3
SiO_2_	6.1 ± 1.0	0.5 ± 0.7
NiO	45.5 ± 16.8**	35.0 ± 17.0***
Co_3_O_4_	104.4 ± 12.4***	93.8 ± 11.3***
Cr_2_O_3_	39.9 ± 12.1**	1.7 ± 1.6
ZnO	16.0 ± 4.1	13.2 ± 5.0*
CuO	40.5 ± 3.6**	36.9 ± 3.8***

The acute lung inflammogenicity data were used from our previously published data [16,17].

Significance versus vehicle control: ^*^p < 0.05, ^**^p < 0.01, ^***^p < 0.001.

**Table 3:** **Comparison of *in vivo *** **lung inflammation data with *in vitro *** **assay data at the same surface area dose (30 cm**^**2**^**/mL) unless otherwise stated**

	**Rat (150 cm**^**2**^**/rat)**	**A549**		**16-HBE**		**THP-1**		**Alveolar mac**		**Differentiated PBMC**			**DifferentiatedTHP-1**^**a**^			**Combined (A549 + THP-1)**^**a**^	**Haemolysis**
	**granulocytes**	**cytotox**^**b**^	**IL-8**	**cytotox**	**IL-8**	**cytotox**	**IL-1β**	**cytotox**	**IL-1β**	**cytotox**	**IL-1β**	**TNF-α**	**cytotox**	**IL-1β**	**TNF-α**	**IL-8**	**% haemolysis**
CeO_2_	+	-	-	-	+	-	-	+	-	-	-	-	-	-	+	-	-
TiO_2_-rutile	-	-	-	-	-	-	-	-	-	-	-	-	-	+	-	-	-
CB	-	-	-	-	+	-	-	-	-	-	-	-	-	-	-	-	-
SiO_2_	-	-	-	-	-	-	-	-	-	-	-	-	-	-	-	-	-
NiO	+	-	-	-	-	-	-	-	-	+	-	-	-	-	-	-	+
Co_3_O_4_	+	-	-	-	-	-	-	+	-	+	-	-	-	-	-	-	-
Cr_2_O_3_	-	-	-	-	-	-	-	+	-	-	-	-	-	-	-	-	-
ZnO	+	+	+	+	+	-	-	+	-	+	+	+	+	-	-	+	N/A
CuO	+	+	+	+	-	+	-	+	+	+	-	-	+	-	-	+	-
Accuracy		6/9	6/9	6/9	5/9	5/9	4/9	7/9	5/9	8/9	5/9	5/9	6/9	3/9	5/9	6/9	5/8
False positive		0/9	0/9	0/9	1/9	0/9	0/9	1/9	0/9	0/9	0/9	0/9	0/9	1/9	0/9	0/9	0/8
False negative		3/9	3/9	3/9	3/9	4/9	5/9	1/9	4/9	1/9	4/9	4/9	3/9	5/9	4/9	3/9	3/8

^a^The highest dose for CuO NP was 3 cm^2^/mL.

^b^Cytotoxicity was measured by lactated dehydrogenase (LDH) or trypan blue exclusion assay.

Minimum four independent experiments were performed for each assay.

Statistically significant changes compared to vehicle control were marked as '+’ and no significant changes were noted as '-’.

**Table 4:** **Comparison of *in vivo *** **lung inflammation data with *in vitro *** **assay data based on any dose even implausibly high ones**

	**Rat (150 cm**^**2**^**/rat)**	**A549**		**16-HBE**		**THP-1**		**Alveolar mac**		**Differentiated PBMC**			**Differentiated THP-1**^**a**^			**Combined (A549 + THP-1)**^**a**^	**Haemolysis**
	**granulocytes**	**cytotox**^**b**^	**IL-8**	**cytotox**	**IL-8**	**cytotox**	**IL-1β**	**cytotox**	**IL-1β**	**cytotox**	**IL-1β**	**TNF-α**	**cytotox**	**IL-1β**	**TNF-α**	**IL-8**	**% haemolysis**
CeO_2_	+	-	-	-	+	-	-	+	-	-	-	-	+	+	+	+	+
TiO_2_-rutile	-	-	-	-	-	-	+	+	+	-	+	+	+	+	+	+	-
CB	-	-	-	-	+	-	-	-	-	+	+	-	-	+	-	-	-
SiO_2_	-	-	-	-	-	-	-	-	-	-	-	-	-	+	-	-	-
NiO	+	-	-	-	-	-	-	+	-	+	+	-	+	-	+	-	+
Co_3_O_4_	+	-	-	-	-	-	-	+	-	+	-	-	-	+	-	-	+
Cr_2_O_3_	-	+	-	-	-	+	-	+	-	+	+	-	+	+	+	+	-
ZnO	+	+	+	+	+	-	-	+	-	+	+	+	+	-	-	+	N/A
CuO	+	+	+	+	-	+	-	+	+	+	-	-	+	-	-	+	+
Accuracy		5/9	6/9	6/9	5/9	4/9	3/9	7/9	4/9	6/9	3/9	4/9	6/9	2/9	4/9	5/9	8/8
False positive		1/9	0/9	0/9	1/9	1/9	1/9	2/9	1/9	2/9	3/9	1/9	2/9	4/9	2/9	2/9	0/8
False negative		3/9	3/9	3/9	3/9	4/9	5/9	0/9	4/9	1/9	3/9	4/9	1/9	3/9	3/9	2/9	0/8

^a^The highest dose for CuO NP was 3 cm^2^/mL.

^b^Cytotoxicity was measured by lactated dehydrogenase (LDH) or trypan blue exclusion assay.

The statistically significant changes compared to vehicle control were marked as '+’ and no significant changes were noted as '-’.

## Discussion

Cell-based *in vitro* toxicity testing is very important not only for evaluation of mechanism of action but also for alternative testing methods to replace animal experimentation [14]. In nanotoxicology, *in vitro* toxicity testing for NPs has a high priority and indeed most of the studies have been conducted *in vitro*[15]. However, the responses to NPs are known to be very variable depending on cell type because of the diverse physiological functions of cells and the heterogeneous physicochemical properties of NPs [8]. In this study, we used 9 predominantly metal oxide NPs and tested cytotoxicity and pro-inflammatory cytokine expression using 8 different cell-based toxicity testing models relevant to the lung. The efficacy of *in vitro* testing methods was evaluated against the potential of the panel to cause acute lung inflammation in rats using data that we published previously [16,17]. All NPs used in *in vivo* and *in vitro* study were exactly same batch and both studies were performed contemporaneously.

Differentiated THP-1 cells by PMA treatment were considerably more sensitive to NPs with respect to both cytotoxicity and cytokine expression, compared to undifferentiated THP-1 cells. The pattern of IL-1β expression was slightly different from that of TNF-α. For example, TiO_2_ NP induced the greatest response with respect to IL-1β but only a slight increase in TNF-α; NiO NP on the other hand did not increase IL-1β but caused a substantial increase in TNF-α levels. This might mean that each metal oxide triggers inflammation by different pathways, possibly resulting in different modes of inflammation depending on their physicochemical properties [16]. Interestingly ZnO and CuO NP failed to induce any cytokines response but were found to be highly cytotoxic. The cytotoxicity of ZnO and CuO NP is likely due to their water soluble ions [18]. In addition to this, accelerated dissolution of ZnO [19] and CuO NP [20] inside of the acidic lysosomal fluid might be more effective in macrophages compared to monocytic cells. Several NPs such as SiO_2_ and TiO_2_ NP were recently known to cause release of IL-1β by activating NLR pyrin domain containing 3 (Nlrp3) inflammasome [21,22]. However, ZnO NP did not activate the Nlrp3 inflammasome and failed to stimulate release of IL-1β and the mechanism of inflammasome activation was poorly understood [21]. The pH-dependent dissolution of ZnO and CuO NP inside phagolysosomes causes the NPs to lose the physico-chemical properties that might influence the phagocytosis-related inflammasome activation and pro-inflammatory cytokine expression. The expression of IL-1β by NPs was markedly reduced by inhibition of phagocytosis using cytochalasin D. Therefore, phagocytosis is an important mechanism of producing IL-1β by activation of inflammasome. TNF-α is another important pro-inflammatory cytokine driving inflammation in the lung and CeO_2_ and NiO NP, the two NPs highly responsive to TNF-α, was consistent with the inflammogenicity in the lung [16].

Treatment of A549 cells with NP-free conditioned medium from THP-1 cells (combined culture) showed much greater response in IL-8 production compared to the direct particle exposure. The increases of IL-8 in A549 cells caused by CeO_2_, TiO_2_, and Cr_2_O_3_ NP in the combined culture were mainly due to the TNF-α and IL-1β released by THP-1 cells. The increased IL-8 levels released by A549 cells on treatment with ZnO and CuO NP may be due to IL-8 produced by THP-1 cells and the release of water soluble metal ions by THP-1 cells. Although the combined culture is a good method to evaluate the action of pro-inflammatory cytokines released from one cell type on another cell type [23-25], the toxic ions released from soluble NPs also should be carefully taken into account in this assay [18].

In our previous studies we reported that the CeO_2_, NiO, and Co_3_O_4_ NP had surface charge as their biologically effective dose (BED) whilst ZnO and CuO NP had soluble toxic ions as their BED [26,27]. When NPs were inhaled, NPs gain a protein corona and so the surface charge might be blocked and the toxicities mitigated [28]. However, the protein corona may have little role with NPs which dissolve in acidic conditions [26]. This might be the reason why the most of *in vitro* assay data of ZnO and CuO NP are consistent with the lung inflammation data.

When NPs were dosed at 30 cm^2^/mL which is the only overlapping dose between NPs, the cytotoxicity in differentiated peripheral blood mononuclear cells was the most consistent with the lung inflammation data. In addition, haemolysis assay showed the best accuracy (100%) when haemolysis at any dose was taken to represent positivity in the assay. The haemolysis assay has been proposed as a good model for prediction of *in vivo* lung inflammation [29]. However, haemolysis assay is not applicable to NPs like ZnO which have high binding affinity with haemoglobin and small-sized well-dispersed NPs which need ultracentrifugation to get rid of NPs. In addition, the haemolysis assay should use protein-free saline as a vehicle because protein corona protects the surface reactivity in cell culture condition [26,30,31]. Although both cell culture and *in vivo* condition can produce a corona, the surface reactivity might be unmasked more fully *in vivo*[26] but unmasking might be variable in *in vitro* depending on the cell types and culture conditions. This might be due to the differences in the enzymatic activity inside of phagolysosomes between each model [32] and synergistic effect by cross-talking between each cell type. Among 8 different *in vitro* systems, alveolar macrophages, differentiated PBMC, and red blood cells were better for prediction of *in vivo* lung inflammogenicity of NPs than other systems. As a minimum set of *in vitro* endpoints, cytotoxicity assay can be suggested for better parameters for correlation with *in vivo* lung inflammogenicity than pro-inflammatory cytokine assay.

## Conclusions

Based on the toxicity mechanisms of NPs, two different approaches can be applied for prediction of *in vivo* lung inflammogenicity. Most *in vitro* assays may detect toxic or inflammogenic potential of NPs if the NPs act via soluble ions (i.e., ZnO and CuO NP). However, *in vitro* assays appear not to be good at detecting metal oxide NPs that act via surface charge as their mechanism of toxicity, with the key exception of haemolysis.

## Materials and methods

### Nanoparticle and characterization

A total of 9 different predominantly metal oxide NPs [CeO_2_, TiO_2_ (rutile form), carbon black (CB), SiO_2_, NiO, Co_3_O_4_, Cr_2_O_3_, CuO, and ZnO] were purchased from commercial sources (Table 1). The primary size of NP was measured by transmission electron microscopy (TEM) (JEM-1200EX II, JEOL, Tokyo, Japan). The surface area (BET, Brunauer-Emmett-Teller) of NPs was measured using a Micromeritics TriStar 3000 (Bedfordshire, UK). Agglomeration status of NP in PBS with/without dispersion medium (fetal bovine serum, FBS) was measured by dynamic light scattering (Brookhaven 90 plus; Holtsville, NY, USA). Endotoxin levels in NP suspensions were evaluated by an endpoint chromogenic Limulus Amebocyte Lysate (LAL) assay (Cambrex, MD, USA). The detection limit of LAL kit was 0.1 – 1.0 EU/ml.

### Rationale for dose selection

All experiments were performed using surface area as a dose metric because surface area is considered to be the most appropriate metric for assessing NP toxicity *in vivo* and *in vitro*[12]. For *in vitro* assays, we performed preliminary dose-ranging studies for all NPs from 0.1 to 300 cm^2^/mL. Based on their cytotoxicity, all NPs were used at doses of 30, 100, and 300 cm^2^/mL, except for CuO and ZnO NPs, which were treated at 3, 10, 30 cm^2^/mL because of their greater toxicity. In our previous studies, NPs were intratracheally instilled into the lungs of female Wistar rats at 50, 150, 250 cm^2^/mL [16,17,29]. As a result, CeO_2_ and NiO NP were inflammogenic from 150 cm^2^/mL and ZnO and CuO NP were inflammogenic from 50 cm^2^/mL. Treatment of TiO_2_, CB, SiO_2_ did not show any significant inflammation. Therefore, we selected 150 cm^2^/mL for acute lung inflammation because NiO NP is well known as toxic particles in human cell lines [33] and in rats [34]. To evaluate the correlation of *in vitro* data against *in vivo* data, the dose for *in vivo* was fixed at 150 cm^2^/mL and compared with *in vitro* data at various doses.

### Dispersion of NPs

Because NPs showed some agglomerates which are not readily dispersed without any stresses (i.e., sonication) in PBS, NPs were dispersed with 5% FBS which provide a protein corona on the surface of particles as previously described method [16].

### Cell culture

When NPs are inhaled, they immediately interact with epithelial cells and then alveolar macrophages. Red blood cells have been used in particle toxicology studies to determine direct membranolytic effects of particle surfaces and we emphasise that interactions with red blood cells are not part of the pathophysiological mechanism by which NP act. Therefore we selected alveolar epithelial cells – A549 cells, bronchial epithelial cells – 16-HBE cells, monocytic/macrophage cells – THP-1 cells, human peripheral blood mononuclear cells (PBMC), and rat alveolar macrophages and human red blood cells. A549 cells were obtained from the European Collection of Animal Cell Cultures and THP-1 cells were purchased from American Type Culture Collection (ATCC). 16-HBE cells originated from Dr. Gruenert of the University of California, San Francisco, USA [35]. Rat alveolar macrophages underwent primary culture following whole lung lavage in 7-week old healthy female Wistar rats. Human peripheral whole blood was collected from healthy consenting volunteers (University of Edinburgh) and PBMC were then isolated from buffy coats according to the previously described method [36]. A549 cells and 16-HBE cells were cultured in DMEM medium containing 5% FBS and THP-1 cells, PBMC, and rat alveolar macrophages were cultured in RPMI-1640 medium containing 10% FBS. Cells were cultured at 37°C with 5% CO_2_, 2 mM L-glutamine (Life Technologies, Paisley, UK), 100 IU/mL penicillin, and 100 U/mL streptomycin (Life Technologies). The number of cells for seeding to 6-well plate was 2 × 10^5^ cells/mL for A549 cells and 16-HBE cells and 1 × 10^6^ cells/mL for monocytic/macrophage cells. The monocytic THP-1 cells were differentiated to macrophages with 10 ng/mL of phorbol myristate acetate (PMA; Sigma-Aldrich, Gillingham, Dorset, UK) for 2 days and Peripheral blood monocyte-derived macrophages (PBMDM) were differentiated from PBMC by culturing for 5 days. After differentiation, cells were washed three times with PBS and NPs were treated.

### Inhibition of phagocytosis by cytochalasin D

To evaluate the role of phagocytosis on the pro-inflammatory cytokine release, we treated cytochalasin D which is a well characterized inhibitor of phagocytosis. After differentiation of THP-1 cells by PMA, 0.2 μM of cytochalasin D (Enzo Life Sciences, Exeter, UK) were added to NP suspensions and treated for 24 h. The cell supernatants were then collected for lactate dehydrogenase (LDH) and cytokine measurement.

### NP treatment and measurement of cytotoxicity

Three different doses of NPs were dispersed in FBS first with the volume of 5% in working solution and cell culture medium was added as described above. NPs were then added to cell cultures and incubated for 24 h. Cytotoxicity was measured using a lactate dehydrogenase (LDH) assay kit (Roche Diagnostics Ltd., Burgess Hill, UK) in the NP-free cell culture supernatant collected by three rounds of centrifugation at 15000 × *g* for 20 min. Vehicle control and 0.1% Triton-X treatment were used as a negative and positive control, respectively. The cytotoxicity of NP was expressed by percentage compared to complete cytotoxicity (0.1% Triton-X). To evaluate the adsorption of LDH on the NPs, bare NPs (NPs alone) or NPs pre-incubated in 5% FBS to give them a protein corona were incubated with A549 cell extract rich in LDH and LDH assay was then performed. Briefly, NPs with or without 5% FBS at the highest doses used in *in vitro* study (30 or 300 cm^2^/mL) were incubated with 0.1% Triton-X treated A549 cells for 30 min at room temperature. LDH assay was performed using NP containing-cell lysate solution according to the instruction manual (Roche Diagnostics Ltd.). After colour development, solutions were centrifuged at 15000 ×g for 20 min to get rid of NPs and measured absorbance at 490 nm. As a result, bare NPs showed variable adsorption with some NP adsorbing LDH, whilst NPs with a protein corona showed minimal adsorption (data not shown). Therefore, the adsorption of LDH on the NP with a corona is minimal in this setting. Some metal ions released from NPs are known to inhibit the color development of LDH assay [37]. In our panel of NPs, trypan blue exclusion test was applied to ZnO and CuO NP owing to this interference.

### Combined cell culture

Because one of the critical components of the inflammatory response induced by NPs in the lung is the release of inflammatory mediators in contact with alveolar macrophages, we treated conditioned medium of THP-1 cells to A549 cells by modification of the previously described method [25]. Briefly, differentiated THP-1 cells by PMA as mentioned above were treated with NPs for 24 h. NP-free supernatant was then prepared by three rounds of centrifugation at 15000 ×*g* for 20 min and treated to A549 cells for 4 h. After 4 h of incubation, medium was washed with PBS for three times and replaced with fresh DMEM medium and incubated for an additional 20 h.

### Measurement of cytokines (IL-1β, IL-8, and TNF-α)

IL-1β, IL-8, and TNF-α are pro-inflammatory cytokines correlated with inflammogenic potential of particles [38]. We measured IL-8 protein levels in the supernatant of A549 cells, 16-HBE cells, and combined culture and IL-1β and TNF-α in supernatants of monocytes/macrophages cells (THP-1 cells, PBMDM, and alveolar macrophages) according to the instruction manual (R&D Systems).

### Haemolysis assay

Haemolysis assay was performed according to the previously described method [29]. Briefly, human red blood cells were incubated with NPs at 30, 100, and 300 cm^2^/mL dispersed in saline without any proteins. Saline and 0.1% Triton X-100 was used as negative and positive control, respectively. After 30 min incubation, the amount of released haemoglobin was determined by absorbance at λ = 550 nm. ZnO NP was excluded in this assay owing to high binding affinity with haemoglobin [26].

### Correlation of *in vitro* assays with *in vivo* acute lung inflammogenicity

To evaluate the correlation of *in vitro* assays with *in vivo* toxicity, we used some previously published animal experimental data from our own group [16,17] in order to avoid unnecessary sacrifice of animals. Although *in vivo* data were published earlier, both studies were performed at the same time with the same batch of NPs. The number of total granulocytes was selected as a marker for acute lung inflammogenicity. For *in vitro* assays, statistically significant increases in any doses were regarded as “positive” and other cases were regarded as “negative” (i.e. no effect). For *in vivo* experiments, statistically significant increases in the number of total granulocytes compared to vehicle control were regarded as “positive”. Because one of the most important parameter comparing each result was the treatment dose, we compared *in vitro* assays at 30 cm^2^/mL (the only overlapping dose used for all the particles except in Figures 6 and 7 where 3 cm^2^/mL was the highest dose for CuO NP) or any dose showing statistical significance.

### Statistical analysis

We conducted minimum 4 individual experiments and all data were expressed as mean ± standard deviations. Data were analyzed using the GraphPad Prism 5 (GraphPad Software, Inc., La Jolla, CA, USA). One-way analysis of variance with post hoc Tukey’s pairwise comparisons test was used to compare the differences between groups. A *p* value of < 0.05 was considered to be statistically significant.

## Competing interests

The authors declare they have no competing financial interests.

## Authors’ contributions

WSC, RD, MB, ILM, WMacN, and KD provided key intellectual input culminating in the conception and design of these studies and aided in the writing of this manuscript. The studies were carried out by WSC who also contributed to the writing of the manuscript. JKL and JJ provided expertise on interpretation of data and both contributed to the writing of the manuscript. All authors read and approved the final manuscript.

## References

[B1] Nel AE Madler L Velegol D Xia T Hoek EM Somasundaran P Klaessig F Castranova V Thompson M Understanding biophysicochemical interactions at the nano-bio interface Nat Mater 2009 10 543 557 10.1038/nmat2442 19525947

[B2] Borm PJ Robbins D Haubold S Kuhlbusch T Fissan H Donaldson K Schins R Stone V Kreyling W Lademann J The potential risks of nanomaterials: a review carried out for ECETOC Part Fibre Toxicol 2006 10 11 10.1186/1743-8977-3-11 16907977 PMC1584248

[B3] Organisation for Economic Co-operation and Development Nanosafety at the OECD: the first five years 2006–2010 2011 Paris: OECD

[B4] Organisation for Economic Co-operation and Development Six years of OECD work on the safety of manufactured nanomaterials: achievements and future opportunities 2013 Paris: OECD

[B5] Donaldson K Borm PJ Castranova V Gulumian M The limits of testing particle-mediated oxidative stress *in vitro* in predicting diverse pathologies; relevance for testing of nanoparticles Part Fibre Toxicol 2009 10 13 10.1186/1743-8977-6-13 19397808 PMC2685764

[B6] Alfaro-Moreno E Nawrot TS Vanaudenaerde BM Hoylaerts MF Vanoirbeek JA Nemery B Hoet PH Co-cultures of multiple cell types mimic pulmonary cell communication in response to urban PM10 Eur Respir J 2008 10 1184 1194 10.1183/09031936.00044008 18653652

[B7] Park EK Jung HS Yang HI Yoo MC Kim C Kim KS Optimized THP-1 differentiation is required for the detection of responses to weak stimuli Inflamm Res 2007 10 45 50 10.1007/s00011-007-6115-5 17334670

[B8] Sohaebuddin SK Thevenot PT Baker D Eaton JW Tang L Nanomaterial cytotoxicity is composition, size, and cell type dependent Part Fibre Toxicol 2010 10 22 10.1186/1743-8977-7-22 20727197 PMC2936333

[B9] Kroll A Dierker C Rommel C Hahn D Wohlleben W Schulze-Isfort C Gobbert C Voetz M Hardinghaus F Schnekenburger J Cytotoxicity screening of 23 engineered nanomaterials using a test matrix of ten cell lines and three different assays Part Fibre Toxicol 2011 10 9 10.1186/1743-8977-8-9 21345205 PMC3059267

[B10] Sayes CM Reed KL Warheit DB Assessing toxicity of fine and nanoparticles: comparing *in vitro* measurements to *in vivo* pulmonary toxicity profiles Toxicol Sci 2007 10 163 180 10.1093/toxsci/kfm018 17301066

[B11] Editorial The dose makes the poison Nat Nanotechnol 2011 10 329 21654642 10.1038/nnano.2011.87

[B12] Duffin R Tran L Brown D Stone V Donaldson K Proinflammogenic effects of low-toxicity and metal nanoparticles *in vivo* and *in vitro*: highlighting the role of particle surface area and surface reactivity Inhal Toxicol 2007 10 849 856 10.1080/08958370701479323 17687716

[B13] Fubini B Ghiazza M Fenoglio I Physico-chemical features of engineered nanoparticles relevant to their toxicity Nanotoxicology 2010 10 347 363 10.3109/17435390.2010.509519 20858045

[B14] Oberdorster G Maynard A Donaldson K Castranova V Fitzpatrick J Ausman K Carter J Karn B Kreyling W Lai D Principles for characterizing the potential human health effects from exposure to nanomaterials: elements of a screening strategy Part Fibre Toxicol 2005 10 8 10.1186/1743-8977-2-8 16209704 PMC1260029

[B15] Johnston HJ Hutchison G Christensen FM Peters S Hankin S Stone V A review of the *in vivo* and *in vitro* toxicity of silver and gold particulates: particle attributes and biological mechanisms responsible for the observed toxicity Crit Rev Toxicol 2010 10 328 346 10.3109/10408440903453074 20128631

[B16] Cho WS Duffin R Poland CA Howie SE MacNee W Bradley M Megson IL Donaldson K Metal oxide nanoparticles induce unique inflammatory footprints in the lung: important implications for nanoparticle testing Environ Health Perspect 2010 10 1699 1706 10.1289/ehp.1002201 20729176 PMC3002189

[B17] Cho WS Duffin R Bradley M Megson IL MacNee W Howie SEM Donaldson K NiO and Co_3_O_4_ nanoparticles induce lung DTH-like responses and alveolar lipoproteinosis Eur Respir J 2012 10 546 557 10.1183/09031936.00047111 21828028

[B18] Cho WS Duffin R Poland CA Duschl A Oostingh GJ Macnee W Bradley M Megson IL Donaldson K Differential pro-inflammatory effects of metal oxide nanoparticles and their soluble ions *in vitro* and *in vivo*; zinc and copper nanoparticles, but not their ions, recruit eosinophils to the lungs Nanotoxicology 2012 10 22 35 10.3109/17435390.2011.552810 21332300

[B19] Bian SW Mudunkotuwa IA Rupasinghe T Grassian VH Aggregation and dissolution of 4 nm ZnO nanoparticles in aqueous environments: influence of pH, ionic strength, size, and adsorption of humic acid Langmuir 2011 10 6059 6068 10.1021/la200570n 21500814

[B20] Studer AM Limbach LK Van Duc L Krumeich F Athanassiou EK Gerber LC Moch H Stark WJ Nanoparticle cytotoxicity depends on intracellular solubility: comparison of stabilized copper metal and degradable copper oxide nanoparticles Toxicol Lett 2010 10 169 174 10.1016/j.toxlet.2010.05.012 20621582

[B21] Yazdi AS Guarda G Riteau N Drexler SK Tardivel A Couillin I Tschopp J Nanoparticles activate the NLR pyrin domain containing 3 (Nlrp3) inflammasome and cause pulmonary inflammation through release of IL-1alpha and IL-1beta Proc Natl Acad Sci USA 2010 10 19449 19454 10.1073/pnas.1008155107 20974980 PMC2984140

[B22] Hornung V Bauernfeind F Halle A Samstad EO Kono H Rock KL Fitzgerald KA Latz E Silica crystals and aluminum salts activate the NALP3 inflammasome through phagosomal destabilization Nat Immunol 2008 10 847 856 10.1038/ni.1631 18604214 PMC2834784

[B23] Totlandsdal AI Refsnes M Skomedal T Osnes JB Schwarze PE Lag M Particle-induced cytokine responses in cardiac cell cultures–the effect of particles versus soluble mediators released by particle-exposed lung cells Toxicol Sci 2008 10 233 241 10.1093/toxsci/kfn162 18700232

[B24] Shaw CA Robertson S Miller MR Duffin R Tabor CM Donaldson K Newby DE Hadoke PW Diesel exhaust particulate-exposed macrophages cause marked endothelial cell activation Am J Respir Cell Mol Biol 2010 10 840 851 20693402 10.1165/rcmb.2010-0011OC

[B25] Jimenez LA Drost EM Gilmour PS Rahman I Antonicelli F Ritchie H MacNee W Donaldson K PM(10)-exposed macrophages stimulate a proinflammatory response in lung epithelial cells via TNF-alpha Am J Physiol Lung Cell Mol Physiol 2002 10 L237 L248 11792628 10.1152/ajplung.00024.2001

[B26] Cho WS Duffin R Thielbeer F Bradley M Megson IL Macnee W Poland CA Tran CL Donaldson K Zeta potential and solubility to toxic ions as mechanisms of lung inflammation caused by metal/metal oxide nanoparticles Toxicol Sci 2012 10 469 477 10.1093/toxsci/kfs006 22240982

[B27] Donaldson K Schinwald A Murphy F Cho WS Duffin R Tran L Poland C The biologically effective dose in inhalation nanotoxicology Acc Chem Res 2013 10 3 723 732 10.1021/ar300092y 23003923

[B28] Thielbeer F Donaldson K Bradley M Johansson EM Cho WS Duffin R Megson IL Macnee W Surface functionalization affects the zeta potential, coronal stability and membranolytic activity of polymeric nanoparticles Nanotoxicologyin press 10.3109/17435390.2013.773465 23379633

[B29] Lu S Duffin R Poland C Daly P Murphy F Drost E Macnee W Stone V Donaldson K Efficacy of simple short-term *in vitro* assays for predicting the potential of metal oxide nanoparticles to cause pulmonary inflammation Environ Health Perspect 2009 10 241 247 19270794 10.1289/ehp.11811 PMC2649226

[B30] Casals E Puntes VF Inorganic nanoparticle biomolecular corona: formation, evolution and biological impact Nanomedicine (Lond) 2012 10 12 1917 1930 10.2217/nnm.12.169 23249335

[B31] Maiorano G Sabella S Sorce B Brunetti V Malvindi MA Cingolani R Pompa PP Effects of cell culture media on the dynamic formation of protein-nanoparticle complexes and influence on the cellular response ACS Nano 2010 10 12 7481 7491 10.1021/nn101557e 21082814

[B32] Macickova T Navarova J Urbancikova M Horakova K Comparison of isoproterenol-induced changes in lysosomal enzyme activity *in vivo* and *in vitro* Gen Physiol Biophys 1999 10 86 91 10703725

[B33] Horie M Nishio K Fujita K Kato H Nakamura A Kinugasa S Endoh S Miyauchi A Yamamoto K Murayama H Ultrafine NiO particles induce cytotoxicity *in vitro* by cellular uptake and subsequent Ni(II) release Chem Res Toxicol 2009 10 1415 1426 10.1021/tx900171n 19630433

[B34] Ogami A Morimoto Y Myojo T Oyabu T Murakami M Todoroki M Nishi K Kadoya C Yamamoto M Tanaka I Pathological features of different sizes of nickel oxide following intratracheal instillation in rats Inhal Toxicol 2009 10 812 818 10.1080/08958370802499022 19225964

[B35] Cozens AL Yezzi MJ Kunzelmann K Ohrui T Chin L Eng K Finkbeiner WE Widdicombe JH Gruenert DC CFTR expression and chloride secretion in polarized immortal human bronchial epithelial cells Am J Respir Cell Mol Biol 1994 10 1 38 47 10.1165/ajrcmb.10.1.7507342 7507342

[B36] Dransfield I Buckle AM Savill JS McDowall A Haslett C Hogg N Neutrophil apoptosis is associated with a reduction in CD16 (Fc gamma RIII) expression J Immunol 1994 10 1254 1263 8027553

[B37] Kroll A Pillukat MH Hahn D Schnekenburger J Current *in vitro* methods in nanoparticle risk assessment: limitations and challenges Eur J Pharm Biopharm 2009 10 370 377 10.1016/j.ejpb.2008.08.009 18775492

[B38] Monteiller C Tran L MacNee W Faux S Jones A Miller B Donaldson K The pro-inflammatory effects of low-toxicity low-solubility particles, nanoparticles and fine particles, on epithelial cells *in vitro*: the role of surface area Occup Environ Med 2007 10 609 615 10.1136/oem.2005.024802 17409182 PMC2092561

